# Case report: Mirror paraclinoid aneurysm associated with mirror clinoidal meningioma

**DOI:** 10.3389/fneur.2024.1355865

**Published:** 2024-02-21

**Authors:** Alexander Feliciano Vilcahuamán Paitán, Frederico de Lima Gibbon, Dmitriy Korotkov, Ileane Camallery Castillo, Ambar E. Riley Moguel, Felipe Pereira Salvagni, Feres Chaddad-Neto

**Affiliations:** ^1^Department of Neurology and Neurosurgery, Universidade Federal de São Paulo, São Paulo, Brazil; ^2^Department of Neurosurgery, Complexo Hospitalar da Santa Casa de Misericórdia de Porto Alegre, Porto Alegre, Brazil; ^3^Department of Neurosurgery, Hospital Beneficência Portuguesa de São Paulo, São Paulo, Brazil

**Keywords:** mirror aneurysm, mirror paraclinoid aneurysm, mirror clinoidal meningioma, mirror meningioma, case report

## Abstract

**Introduction:**

Clinoidal meningiomas and paraclinoid aneurysms are individually uncommon, with the coexistence of mirror paraclinoid aneurysms and clinoidal meningiomas presenting an even rarer scenario. While the association between meningiomas and aneurysms is documented, the simultaneous presence of mirror lesions for both pathologies is not reported in the literature.

**Clinical presentation:**

We report a 62-year-old female with a three-month history of moderate bifrontal headaches. Magnetic Resonance Angiography (MRA) revealed mirror paraclinoid aneurysms, prompting surgical intervention. During the procedure, mirror clinoidal meningiomas were incidentally discovered. The left aneurysm was addressed first due to higher rupture risk, followed by the right aneurysm 3 months later. Both meningiomas were confirmed as Transitional Meningiomas (Grade 1; OMS, 2021). The aneurysms were successfully clipped, and the patient had an excellent postoperative outcome.

**Conclusion:**

This case represents a unique occurrence of mirror ophthalmic segment internal carotid artery aneurysms associated with mirror clinoidal meningiomas, a combination not previously reported. Despite the limitations of MRA in detecting small meningiomas, it remains a valuable non-invasive screening tool for neurovascular diseases. The case underscores the need for further research to elucidate the association between cerebral aneurysms and meningiomas.

## 1 Introduction

Clinoidal meningiomas and paraclinoid aneurysms alone are uncommon pathologies. Mirror paraclinoid aneurysms, as well as mirror meningiomas, are even rarer presentations of these diseases. However, although rare, this association between meningioma and aneurysm is already well-established in the literature (1, 2). We present a patient with a mirror paraclinoid aneurysm associated with mirror clinoidal meningiomas, and we found no cases reported in the literature with both pathologies in association.

## 2 Clinical presentation

**Figure 1:**
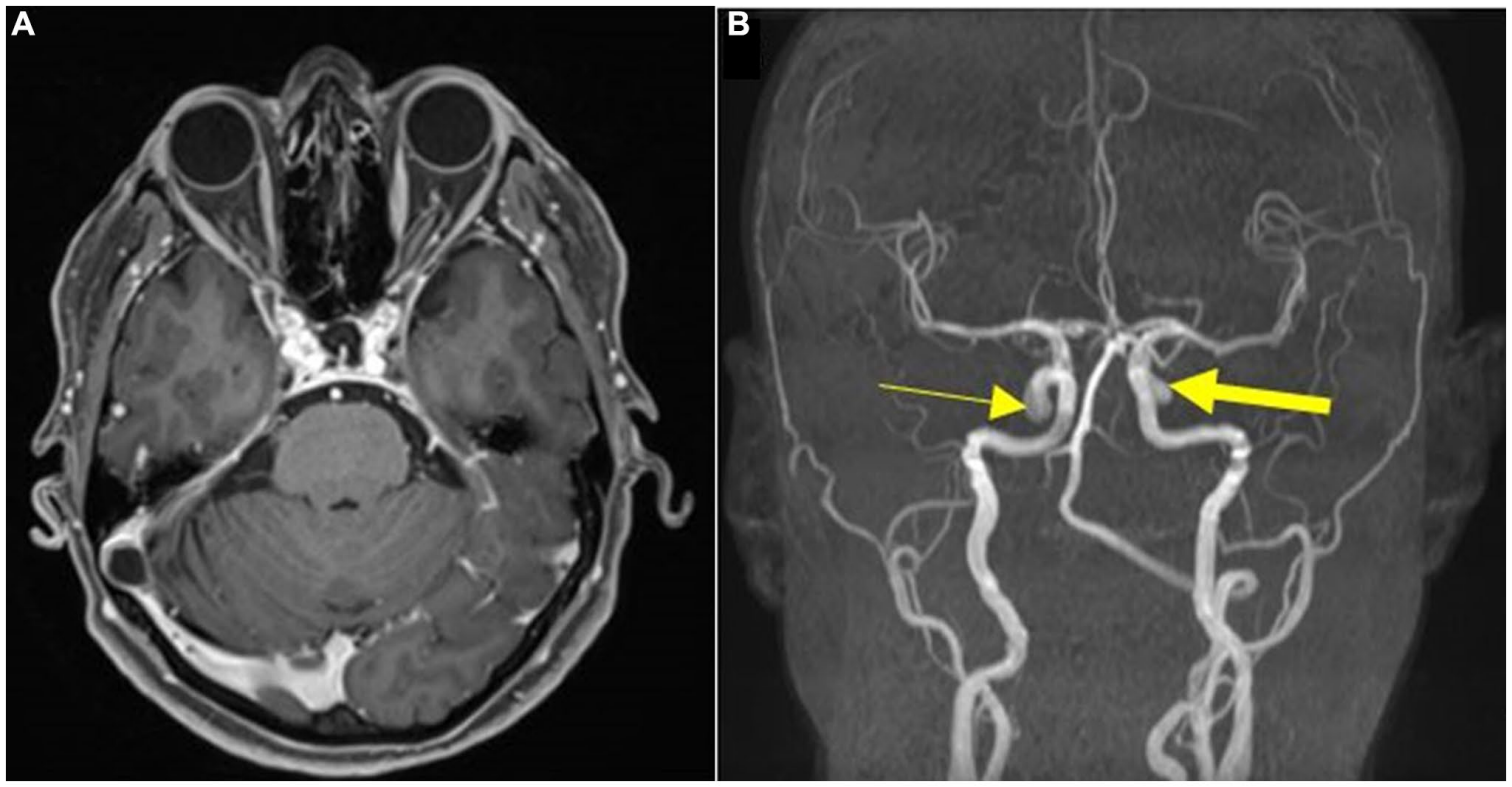
**(A)** Preoperative post gadolinium MRI T1w axial showing paraclinoid mirror aneurysms. **(B)** MRA revealing a right-sided paraclinoid aneurysm (thin arrow) and a left-sided paraclinoid aneurysm (thick arrow).

**Figure 2:**
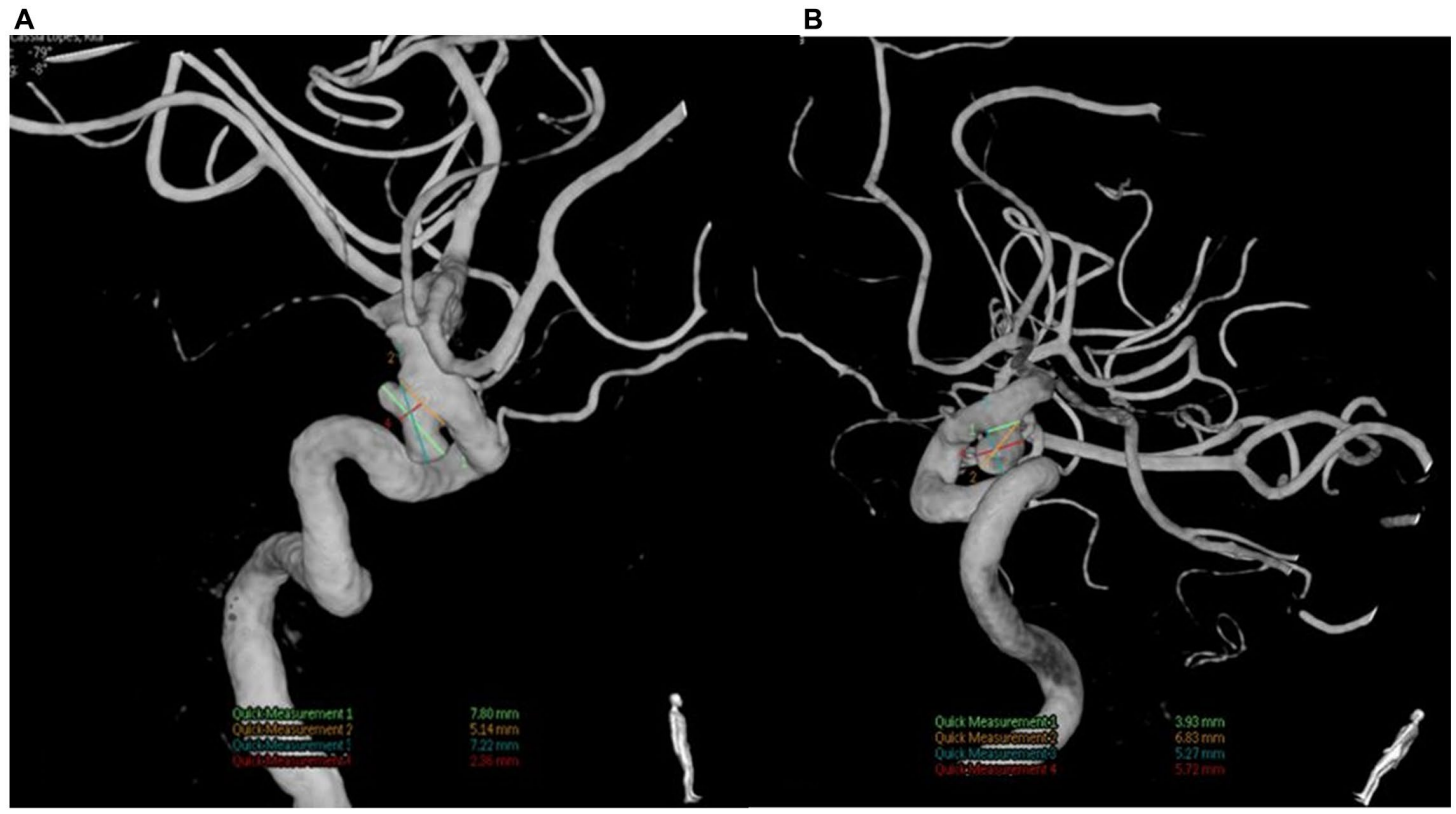
**(A)** Left paraclinoid aneurysm bilobed with a lateral and inferior projection. **(B)** Right paraclinoid aneurysm with inferior projection.

A 62-year-old female presented a moderately intense headache in the bifrontal region as her primary concern. The pain started approximately 3 months ago, improving partially with analgesics, but after the effects of the medication had passed, the pain returned. The patient was previously healthy and had no medical history, relevant family disorders, or chronic or psychological diseases. In addition, the patient had no prior surgical intervention, exposure to radiation, or known genetic disease. Physical and neurological examination was normal. Due to the sudden onset of the headache, a Magnetic Resonance Angiography (MRA) was requested. The MRA revealed two paraclinoid mirror aneurysms (Figure 1), and surgical intervention was indicated after a thorough analysis of the aneurysms, including dome projection, size, and location. The patient was extremely cultured and had already researched about aneurysm treatment techniques. She said she sought the main author’s experience treating this pathology through open surgery. To complement the investigation and surgical planning, digital subtraction angiography (DSA) was requested (Figure 2).

**Figure 3:**
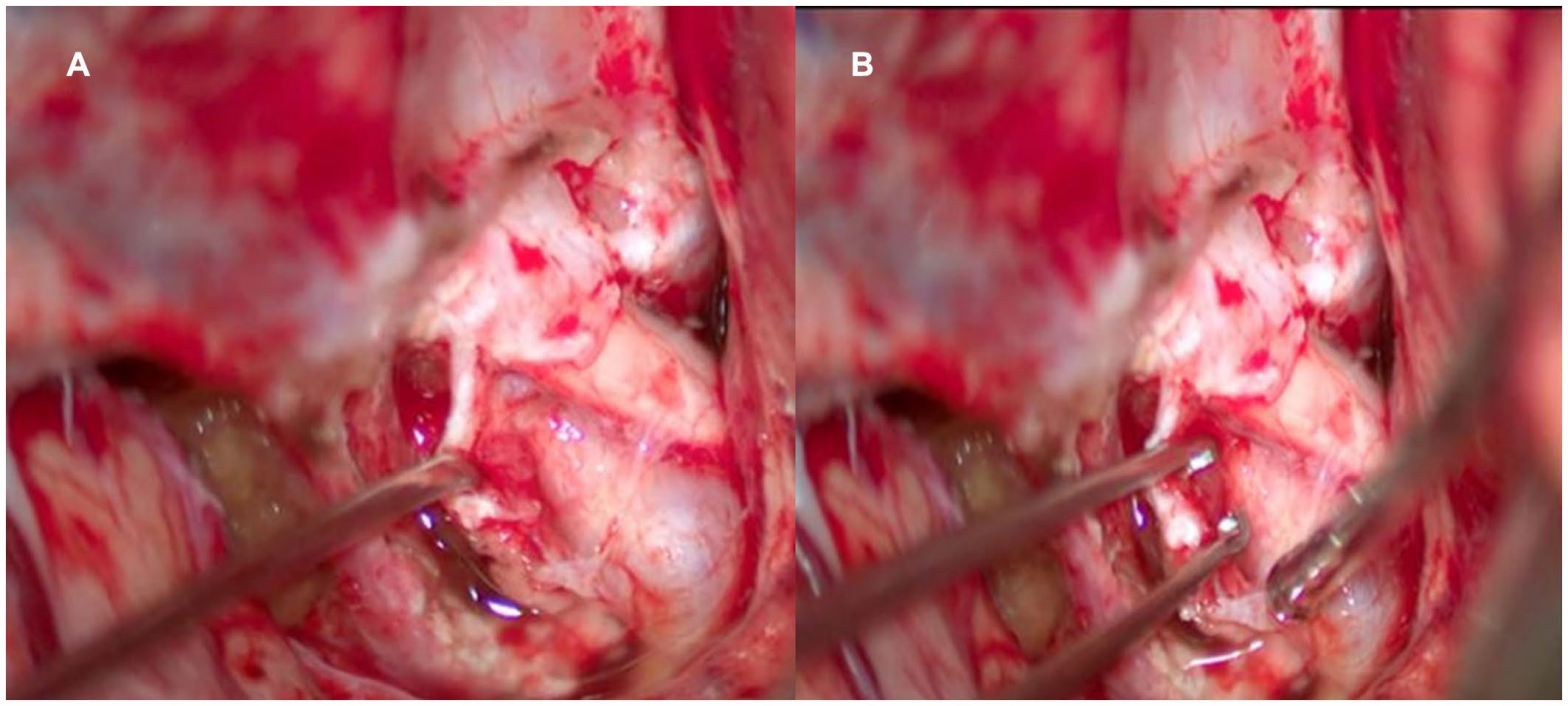
Intraoperative photo of the left optic canal after clineidectomy. **(A)** We appreciate the relation of the tumor in the optic canal. **(B)** Biopsy is performed for histopathological diagnosis.

The exam shows two ventral aneurysms. The left-sided aneurysm was operated first due to its higher rupture risk. It was larger and projected medially, while the aneurysm from the right was smaller and projected inferiorly. During the surgical procedure, when performing the clinoidectomy, a small expansive lesion was observed along the optic canal. The lesion had the appearance of a meningioma (Figure 3). The tumor was easily accessible and was then completely removed with resection from its implantation base (Simpson grade 1) (3).

**Figure 4:**
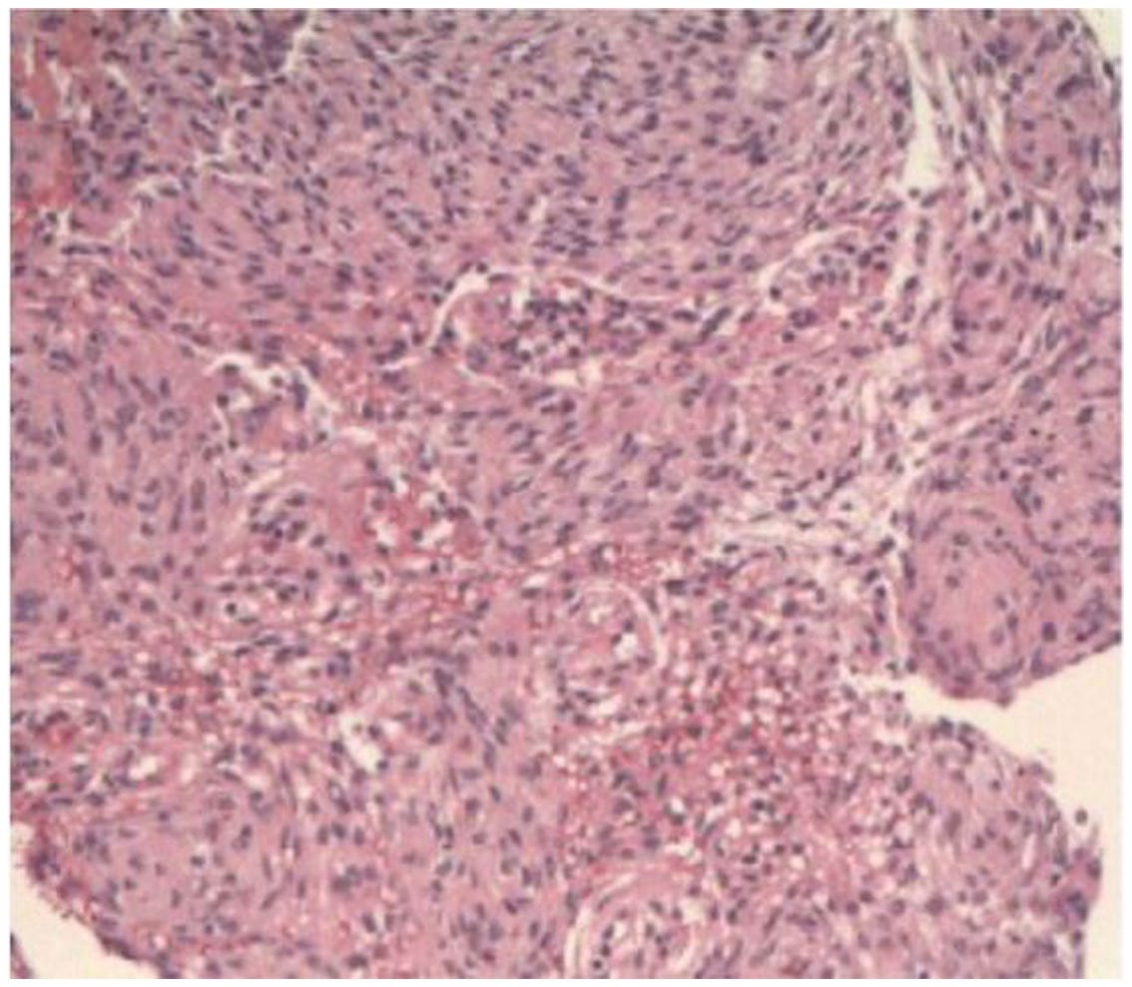
H&E histological photo showing a Transitional Meningioma (Grade 1; OMS 2021).

Later, an anatomopathological analysis biopsy confirmed Transitional Meningioma (Grade 1; OMS, 2021). Surgery and clipping of the aneurysm were uneventful, and the patient did well postoperatively. Approximately 3 months later, surgical intervention was scheduled for the right paraclinoid aneurysm. During the procedure, again while performing the clinoidectomy, an expansive lesion was identified along the optic canal, which was completely resected (Simpson grade 1) and sent for anatomopathological analysis, which surprisingly also confirmed a Transitional Meningioma (Grade 1; OMS, 2021) (Figure 4). Clipping of the aneurysm was performed without complications, and the patient had an excellent postoperative period (modified Ranking Scale 0). No visual impairment or any other deficits were reported by the patient after the procedures. The patient consented to the procedure and to the publication of his/her image.

## 3 Discussion

Mirror aneurysms are a rare subtype of aneurysms, and their incidence varies between 5 and 30% among patients with multiple aneurysms (4, 5). The most common location is in the middle cerebral artery, being uncommon in the internal carotid artery in the paraclinoid region (4, 5). Some factors are associated with a higher incidence of mirror aneurysms, such as smoking and lack of estrogen, but it is not yet known precisely what their roles are in forming mirror aneurysms (4, 6). Our patient was not a smoker, but she was in menopause, which may have been a risk factor for mirror aneurysms.

Meningiomas are the most common benign primary tumors of the central nervous system. They generally present as solitary lesions. However, 10% can be multiple, and mirror meningiomas are extremely rare (7–9). The latter are typically associated with type 2 neurofibromatosis or exposure to radiation (9–11). Our patient did not present any of these risk factors, showing that there are undoubtedly other factors that have not been identified.

Most meningiomas affecting the optic canal are secondary meningiomas, originating from other regions, such as the tuberculum sellae or cavernous sinus, with primary meningiomas of the optic canal being less common. Meningiomas of the optic canal can have two diametrically opposite presentations: either they present early with visual loss, or they are asymptomatic because the size does not cause optic nerve compression. The latter presentation is how our patient behave. In our clinical case, both meningiomas were clinoidal according to the Al-Mefty Group III Classification (12), and due to their size, not even a 3-Tesla MRI was able to identify the tumor. Even though the MRI plays an important role in evaluating tumors with extension for the optical canal size, it is still essential in diagnosis (13). Constructive interference in steady state (CISS) and contrast-enhanced T1-weighted volume-interpolated breath-hold examination (VIBE) sequences are associated with reasonable accuracy in predicting tumor extension to the optic canal (14). However, these sequences are only sometimes requested or routine in our service. Unfortunately, we will not know if these sequences could identify the tumor in our case.

Brain tumors and intracranial aneurysms are rare diseases, and the coexistence of both is even rarer. However, Kim et al. (2) have already shown through a case–control study that the association between meningioma and cerebral aneurysm is well established and that the prevalence of intracranial aneurysm in patients with meningioma is higher than in the general population. The real incidence of the association between brain tumors and intracranial aneurysm is unknown. However, it is known that meningioma is the most associated tumor (1, 15).

Many cases in the literature are case reports in which a brain tumor was the principal diagnosis, and during surgery, the intracranial aneurysm was identified. Our case reports the opposite; here, the primary objective was the treatment of the aneurysm, with the tumor being an incidental finding. In patients who present with a tumor and aneurysms, the patients generally present symptoms related to the tumor and none because of the aneurysm (1). In this case, initial symptoms were probably due to the aneurysm since the meningioma was so small that it could not be identified by Magnetic Resonance Imaging (MRI).

Pia et al. (1) identified a more significant association between meningiomas of the skull base and aneurysms of the internal carotid artery. In contrast, anterior and middle cerebral artery aneurysms are more associated with convexity meningiomas. This is precisely in line with our case since our patient had two clinoidal meningiomas and two internal carotid artery aneurysms.

There are some hypotheses that the tumor could develop a disturbance in local blood flow, which could be responsible for the aneurysm formation (2). However, our case goes against this theory because both meningiomas were so small that they would not be capable of causing significant flow disturbances that would justify the formation of the aneurysm. In this case, certain genetic or systemic factors might be responsible for this association.

Furthermore, paraclinoid aneurysms can be classified according to the Lawton classification (16). Our clinical case presents two aneurysms, which are both classified as ventral paraclinoid aneurysms (16). Endovascular treatment is one of the first therapeutic options for paraclinoid aneurysms, mainly due to its complex surgical approach, excellent catheter access, and constant technological advances in novel devices (17, 18). However, the endovascular technique cannot be used to treat all aneurysms. In some cases, it is associated with lower occlusion rates and greater recurrence than microsurgery (19). Flow-diverting devices are an excellent artifice for the endovascular technique but are still inferior to microsurgery in our clinical practice. Furthermore, based on the experience and results of the senior neurosurgeon who evaluated the characteristics of the aneurismal, such as its morphology, the right aneurysm is saccular, while the left one is bilobed, due to their size both are medium, the projection of the aneurysms are ventral, not presenting comorbidities and the desire of the patient to undergo microsurgery, decided microsurgical treatment.

Additionally, both clinoid meningiomas were type 3 in this case, according to the Al-Mefty classification (12). An essential fact, in this case, is the need to pay extra attention while performing clinoidectomy in these types of aneurysms since the tumor can make the procedure difficult to perform and hurt safety.

Stereotaxic radiosurgery (SRS), such as the Gamma Knife, is also an alternative to treat skull base meningiomas, such as clinoidal meningiomas and cavernous sinus meningiomas (13). The rule of the SRS in treating meningiomas or reducing their recurrences is well established, especially if the involvement of the vessels and cranial nerves or total resection is not performed (20, 21). However, SRS is not an indolent procedure, especially regarding the risk of radiation-induced optic neuropathy (21, 22). In addition, the benefit of the SRS for small meningiomas where Simpson grade 1 is accomplished is questionable since the disease control rate is relatively high without any other adjuvant treatment. In our case, the meningioma was in a very accessible location, where a Simpson grade 1 was accomplished. Therefore, it is unnecessary to carry out any adjuvant treatment, and we only perform radiological control. If tumor recurrence is identified at some point, we usually re-operate the tumor whenever possible, indicating SRS only in selected cases, such as malignant meningiomas or inoperable tumors.

The importance of this article is that many times, we do not make the diagnosis of group III clinoidal meningioma due to their size, which is small and may not cause visual disturbance and may not be observed on MRI. We present the only case of mirror clinoidal meningiomas with mirror paraclinoid aneurysms reported because it was a finding in the surgical act, but there may be more cases if an adequate presurgical study is carried out and confirmed in the operative act. It also demonstrates the importance of having skill in microsurgery for this type of aneurysm since if it were due to the endovascular technique could not diagnose meningiomas of the optic canal, which is crucial because the natural progression of the disease is an inexorable visual deterioration and the results of surgical decompression in the later stages is poor.

## 4 Conclusion

Our case highlights the only case in the literature in which the association between mirror ophthalmic segment internal carotid artery aneurysms and mirror clinoidal meningiomas was identified. Furthermore, MRA can be used as the screening test for patients with non-specific neurological complaints, as it is non-invasive and carries a high specificity and sensitivity for identifying neoplastic and cerebrovascular diseases, even though it failed to identify the meningiomas in our case because of the size. In addition, this case aims to show that more investigations and the subsidy to perform this scientific research are needed to reveal the association between cerebral aneurysms and meningiomas.

Additionally, our case is an example of the versatility of microsurgical treatment since surely treated two brain aneurysms and two brain tumors. If the patient had performed endovascular treatment, even if he could get the same outcome, she would still present two tumors, which would probably have some bad repercussions in the future.

## Data availability statement

The original contributions presented in the study are included in the article/supplementary material, further inquiries can be directed to the corresponding author.

## Ethics statement

The studies involving humans were approved by Ethics and Research Committee of the Federal University of São Paulo. The studies were conducted in accordance with the local legislation and institutional requirements. The participants provided their written informed consent to participate in this study. Written informed consent was obtained from the individual(s) for the publication of any potentially identifiable images or data included in this article.

## Author contributions

AP: Conceptualization, Investigation, Writing – original draft, Writing – review & editing. FG: Investigation, Methodology, Writing – review & editing. DK: Data curation, Writing – review & editing. IC: Data curation, Writing – review & editing. AM: Investigation, Writing – review & editing. FS: Investigation, Writing – review & editing. FC-N: Project administration, Supervision, Validation, Writing – review & editing.
